# Impact of embryo storage time after vitrification on pregnancy and implantation rates

**DOI:** 10.5935/1518-0557.20250053

**Published:** 2025

**Authors:** Rafaela Marra Santana Costa, Renata de Lima Bossi, Márcia Mendonça Carneiro, Marcos Sampaio, Selmo Geber

**Affiliations:** 1 Department of Obstetrics and Gynecology, Federal University of Minas Gerais, Belo Horizonte, Brazil; 2 Clínica Origen, Belo Horizonte, Brazil

**Keywords:** embryo vitrification, thawing, ART, cryopreservation, cryostorage

## Abstract

**Objective:**

The study aimed to assess whether the storage time after vitrification affects clinical pregnancy and implantation rates.

**Methods:**

This multicentric retrospective study involved 1568 cycles of autologous frozen embryo transfers between 2015 and 2019. The patients were divided into groups based on storage time to assess its impact on pregnancy and implantation rates. The variables analyzed were maternal age, cryopreservation stage, and embryo transfer” stage. Logistic regression was used to investigate associations between storage duration, patient age, and transfer outcomes. The study was approved by the Research Ethics Committee of UFMG and informed written consent was waivered.

**Results:**

The length of embryo storage showed an inverse association with pregnancy and implantation rates, correlating with patient age and embryo stage at the time of transfer. Patients aged 36 to 40, and 41 years or older had lower rates of clinical pregnancy compared to those under 35 years of age. Additionally, vitrified embryos transferred at the cleavage stage demonstrated lower rates of clinical pregnancy and implantation compared to those at the blastocyst stage. These results suggest that storage duration, patient age, and embryo stage significantly influence frozen embryo transfer (FET) outcomes.

**Conclusions:**

Storage duration, patient age, and embryo stage can affect FET outcomes, with older women and embryos at the cleavage stage, possibly experiencing lower pregnancy and implantation rates after extended storage.

## INTRODUCTION

Advances in human assisted reproduction, especially in hormonal stimulation protocols and embryo culture, have led to the generation of a substantial number of high-quality embryos. Consequently, the implementation of cryopreservation techniques has become critical for the effective storage of surplus embryos (Kuwayama *et al.*, 2005; Schoolcraft *et al.*, 2011; Niederberger *et al.*, 2018).

Efficient cryopreservation of gametes and embryos has become essential in assisted reproductive treatments, enhancing the safety and efficacy of the procedures. Elective embryo freezing and transfer in subsequent cycles has increased, which is crucial in cases of inadequate endometrial development, asynchrony between egg donors and recipients, the risk of ovarian hyperstimulation syndrome and early elevation of progesterone levels. Additionally, cryopreservation is indicated for fertility preservation or when embryo genetic analysis is indicated. Freezing eliminates the need for new hormonal stimulation and egg collection, allowing transfer of thawed embryos in a new menstrual cycle (Schoolcraft *et al.*, 2011; Niederberger *et al.*, 2018; Ueno *et al.*, 2018; Roque *et al.*, 2019).

Since the first report of pregnancy following the transfer of a cryopreserved embryo in 1983, various cryopreservation techniques have been developed, utilizing different types and concentrations of cryoprotectants, as well as diverse freezing and thawing protocols. The two most common methods are slow freezing and vitrification. Slow freezing involves a gradual decrease in temperature with low concentrations of cryoprotectants, minimizing intracellular ice formation, making it a time-consuming and costly method. Currently, vitrification is the most used technique due to its speed, lack of specific equipment requirements, better embryo survival rates and improved implantation and pregnancy rates (Trounson & Mohr, 1983; Kattera & Chen, 2006; Edgar & Gook, 2012).

Vitrification prevents the formation of ice crystals by solidifying the cells in a vitreous state, using high concentrations of cryoprotectants for cellular dehydration and rapid cooling. Since 1992, vitrification has proven efficient, with variations in protocols according to the type, concentration, volume of cryoprotectants, cooling and thawing times, exposure duration and vitrification systems. In the vitrification process, selected embryos are sequentially placed in increasing concentrations of cryoprotectant droplets to replace water. The exposure time in each droplet is progressively reduced to avoid toxic effects from the cryoprotectants. Subsequently, the embryos are transferred to a vitrification device and immediately submerged in liquid nitrogen (Mukaida *et al.*, 2003; Edgar & Gook, 2012; Wong *et al.*, 2014).

Recently, there has been a significant increase in the number of frozen-thawed embryo transfer cycles. Data from the Latin American Registry of Assisted Reproduction (REDLARA) showed a continuous growth in these cycles over the years, highlighting an increasing proportion of frozen-thawed embryo transfers compared to fresh transfers in clinics across Latin America (Zegers-Hochschild *et al.*, 2025). Similarly, the European Society of Human Reproduction and Embryology (ESHRE) reported this upward trend between 2015 and 2016. Furthermore, a joint report by American Society for Reproductive Medicine (ASRM), Centers for Disease Control and Prevention CDC) and Society for Assisted Reproductive Technology (SART) CDC, and SART, based on data from clinics in the USA, showed that frozen-thawed embryo transfers in 2018 were substantially more frequent than those of fresh embryos from fresh oocytes and frozen oocytes (CDC, 2020; European IVF-monitoring Consortium for ESHRE *et al.*, 2020; Zegers-Hochschild *et al.*, 2025).

Although the vitrification technique is now well established, controversy remains over whether the duration of time an embryo remains frozen affects its viability, implantation and pregnancy rates. Moreover, there is a scarcity of studies investigating how storage time affects embryo survival and pregnancy outcomes (Ueno *et al.*, 2018; Li *et al.*, 2020; Matorras *et al.*, 2021). Accordingly, the aim of this study was to evaluate whether the duration of embryo storage after vitrification impacts implantation and clinical pregnancy rates.

## MATERIAL AND METHODS

A retrospective multicentric study was conducted analyzing data from the medical records of patients who had thawed embryos transferred from January 2015 to December 2019 at three private assisted reproduction centers in Brazil. The study was approved by the National Research Ethics Committee (CAAE: 39343920.8.0000.5149).

The study included 1,568 women who underwent embryo transfer cycles with thawed embryos using autologous oocytes at the blastocyst stage (day 05) or cleavage stage (Day 2- D2; and Day 3 D3). The inclusion criteria comprised patients over 18 years of age who had surplus embryos, frozen using an open vitrification system who then underwent a frozen-thaw cycle on a later date. Each woman was included only once in the study.

Patients were excluded from the study if they did not have any viable embryos available for transfer post-thawing, if they had embryos assessed genetically (PGT - pre-implantation genetic testing), if they received donated embryos and/or oocytes, if they utilized a surrogate uterus, if they presented with severe male factor (< 1 million/ml), if they transferred revitrified embryos or if the embryos underwent extended culture following thawing (thawed embryos on day 02 and transferred on day 03, thawed embryos on day 02 and transferred on day 05, and thawed embryos on day 03 and transferred on day 05). Additionally, patients who underwent more than one embryo transfer of thawed embryos were also excluded.

Ovarian stimulation and follicular aspiration were similar across the three centers, following either the GnRH agonist or antagonist protocol. In the GnRH antagonist protocol, the patients received recombinant gonadotropins (Gonal F, Merck Serono Brazil or Pergoveris, Merck Serono Brazil), starting on the second or third day of the menstrual cycle, with doses adjusted according to age and individual ovarian response, ranging from 75 to 450 IU per day. When the follicles reached a diameter of 14 mm, GnRH antagonist (Cetrodite, Merck Serono Brazil) was administered to block pituitary function until oocyte maturation induction (Colodetti *et al.*, 2020).

In the GnRH agonist protocol, pituitary suppression was achieved using leuprorelin acetate (Lupron, Novartis Brazil), 40 mcg, twice daily, from the second or third day of the menstrual cycle in combination with gonadotropins. Oocyte maturation was triggered with recombinant human chorionic gonadotropin (hCG, Ovidrel Merck Serono Brazil) when at least two follicles reached an average diameter of 17 mm (Colodetti *et al.*, 2020).

The patients underwent ultrasound-guided follicular aspiration 34 to 36 hours after hCG administration under intravenous analgesia. The follicular fluid was sent to the laboratory for identification of the cumulus-oophorus complex and subsequent oocyte identification. Oocyte maturity was assessed two hours later, and those in the metaphase II (MII) stage were inseminated via ICSI. Semen was prepared using a discontinuous gradient (Cook, Australia).

Fertilization was confirmed by identifying two pronuclei 18-20 hours after ICSI. Embryos were then evaluated on the second, third, and/or fifth day of development for morphological grading and subsequent transfer to the uterus and/or freezing of surplus embryos. Embryos kept until day 3 were cultured in Cleavage medium (Cook, Australia), and those maintained from day 3 to day 5 were cultured in Blastocyst medium (Cook, Australia). Culturing was carried out in incubators (Thermo Scientific, USA) at 37°C in 6% CO2.

Cleavage-stage embryos (Day 2 and Day 3) were classified according to the model described by Veeck (1999). Grade I and II embryos were classified as good quality and cryopreserved by vitrification or maintained in culture for subsequent freezing until the blastocyst stage, when they were graded according to the model described by Gardner & Schoolcraft (1999). Good-quality blastocysts (AA, AB, BA, and BB) were vitrified.

Vitrification was performed using a commercial vitrification kit (Ingamed, Brazil). For the thawing process of vitrified embryos, the thawing kit (Ingamed, Brazil) was used according to the manufacturer’s protocol.

Endometrial preparation for the transfer of thawed embryos was carried out in either a natural or artificial cycle. The natural cycle was indicated for patients with regular menstrual cycles. Monitoring was performed by ultrasound on alternate days, starting from the second day of the menstrual cycle, until a follicle reached 17mm in diameter and the endometrium showed a thickness greater than 7 mm. Ovulation was induced with recombinant hCG (Ovidrel - Merck, Brazil), and vaginal progesterone was used for luteal phase support (Utrogestan 100 mg every 8 hours or Crinone 8% once a day) until the tenth week of pregnancy. Progesterone was started 36 hours after hCG administration.

The artificial cycle was indicated for patients with irregular menstrual cycles, who were subjected to pituitary suppression with subcutaneous administration of 3.75 mg leuprorelin (Lectrum) on the second or twenty-first day of the cycle. Suppression was confirmed by vaginal ultrasound and estradiol serum levels. When the endometrium showed a thickness of less than 5 mm and estradiol serum levels were below 50 µg/ml, patients were considered eligible to start estrogen. Estradiol valerate (Primogyna, Bayer, Brazil) was used at a dose of 2 mg/day from the first to fifth day, 4 mg/day from the sixth to tenth day, and 6 mg/day from the eleventh day onward. When the endometrium was thicker than 7 mm and estradiol serum levels exceeded 150 pg/ml, patients were instructed to start vaginal progesterone.

Embryo transfer was performed using the Sydney catheter (Cook, Australia) in a 50 µL drop of medium according to the culture stage, Cleavage or Blastocyst (Cook, Australia), with a 1 mL syringe. Embryo transfer was performed with the patient in the lithotomy position, using a speculum to visualize the cervix and cleanse the vaginal cavity. The transfer catheter was inserted through the external cervical os into the upper third of the endometrial cavity, where the embryos were gently deposited by pressure on the syringe plunger. The procedure was ultrasound-guided and medication was maintained until the day of the pregnancy test. In case of pregnancy confirmation, progesterone was continued until at least the third month of pregnancy.

Pregnancy was confirmed by serum β-hCG measurement on days 9, 11, or 12 after embryo transfer, depending on the embryonic stage at the time of transfer (Day 05, Day 03, or Day 02, respectively). Clinical pregnancy was confirmed by vaginal ultrasound showing at least one gestational sac and the presence of an embryonic heartbeat.

Patients were allocated into four groups according to the freezing time:

- 0 to 90 days before embryo transfer;
- 91 to 180 days before embryo transfer;
- 181 to 360 days before embryo transfer;
- More than 360 days before embryo transfer.

Each group was further subdivided by age group at the time of embryo freezing (up to 35 years, 36-40 years, and 41 years or older) and embryonic development stage: cleavage (D2 or D3) or blastocyst (D5). The implantation rate was calculated as the number of gestational sacs divided by the number of embryos transferred for each patient.

### Statistical Analysis

Sample size was calculated to test the difference in embryo storage time between cases with and without clinical pregnancy. Considering the Wilcoxon Mann-Whitney test, with a 5% significance level, minimum power of 80%, and a minimum effect size of 0.2, at least 412 women who became pregnant and 412 who did not would be required. The calculation was made using the G*Power 3.1.9.4 software.

For analysis of the influence of freezing time on pregnancy rate, a univariate binary logistic regression model was used. To assess the relationship with age and embryo transfer stage, a multiple binary logistic regression model was employed to identify which variable(s) jointly influenced pregnancy occurrence.

A multiple linear regression model was used to analyze the impact of the freezing time’s impact on the implantation rate and its relationship with age and embryo transfer stage. Qualitative variables were represented by frequencies, and quantitative variables by mean ± standard deviation (median). Normality of quantitative variables was assessed using the Shapiro-Wilk test. The Chi-square test was used to evaluate associations between qualitative variables. The Wilcoxon Mann-Whitney test was employed to compare quantitative variables between the two groups.

Results were presented as odds ratios (OR) with 95% confidence intervals (CI 95%). Model fit quality was assessed using the Hosmer-Lemeshow test. All results were considered significant for a *p*-value lower than 5% (*p*<0.05).

## RESULTS

**Graph 1:**
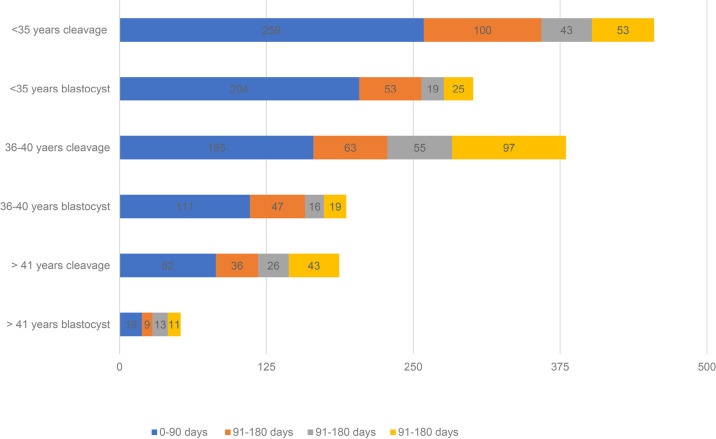
Age and stage of embryo transfer with different storage times.

This study included 1,568 women who underwent their first cycle of frozen embryo transfer (without prior transfer) using autologous oocytes, with embryos frozen through vitrification using an open system between January 2015 and December 2019 at three private assisted reproduction centers in Brazil. The patients were grouped according to storage duration: group 1 (0-90 days: 840 patients), group 2 (91-180 days: 308 patients), group 3 (181-360 days: 172 patients), and group 4 (more than 360 days: 248 patients) (Graph 1).

Among these patients, 757 were under 35 years old, 573 were between 36 and 40 years old, and 238 were 41 years or older. Of the transfers, 1,022 were performed at the cleavage stage, and 546 at the blastocyst stage. A total of 612 women (39.0%) became pregnant. The average number of embryos transferred was 1.96 0.71 (Table 1).

**Table 1:** Characterization of the general sample and according to the occurrence of clinical pregnancy.

Variable	PREGNANCY			
	Sample (n=1568)	Yes (n=612)	No (n=956)	p-value
	n (%)	n (%)	n (%)	
AGE RANGE ≤ 35 years From 36-40 years ≥41 years	757 (48.3) 573 (36.5) 238 (15.2)	324 (52.9) 213 (34.8) 75 (12.3)	433 (45.3) 360 (37.7) 163 (17.1)	0.004^Q^
**EMBRYO TRANSFER STAGE** Cleavage Blastocyst	1.022 (65.2) 546 (34.8)	350 (57.2) 262 (42.8)	672 (70.3) 284 (29.7)	**<0.001^Q^**
**FREEZING TIME** 0 to 90 days 91 to 180 days 181 to 360 days > 360 days	844 (53.8) 304 (19.4) 172 (11.0) 248 (15.8)	362 (59.2) 103 (16.8) 64 (10.5) 83 (13.6)	482 (50.4) 201 (21.0) 08 (11.3) 165 (17.3)	**0.007^Q^**
**Number of embryos transfer**	1.96±0.71 [2.00]	2.11±0.75 [2.00]	1.87±0.66 [2.00]	**<0.001^W^**

Q Chi-square test,

W Wilcoxon Mann-Whitney test.

Patient stratification by storage duration, age and transfer stage can be seen in Graph 1 and Table 1.

### Factors associated with pregnancy rates

Embryos with a storage duration of 91 to 180 days showed lower clinical pregnancy rates compared to those frozen for less than 90 days (OR 0.668, 95% CI 0.500; 0.888).

A higher number of embryos transferred per attempt was associated with an increased likelihood of pregnancy (OR 2.737, 95% CI 2.273; 3.313), considering that each participant underwent only a single embryo transfer.

Women aged 36 to 40 and those of 41 years or older had lower clinical pregnancy rates compared to those aged 35 or younger (OR 0.563, 95% CI 0.438; 0.721 for those aged 36 to 40, and OR 0.319; 95% CI 0.218; 0.462 for women over 41). However, no statistically significant difference in the clinical pregnancy rate was observed between patients aged 36 to 40 years and those aged 41 years or older.

Embryos transferred at the cleavage stage had lower clinical pregnancy rates compared to embryos transferred at the blastocyst stage (OR 0.381, 95% CI 0.298; 0.485).

The model was well-adjusted, as indicated by the p-value of the Hosmer-Lemeshow test >0.05 (Table 2).

**Table 2:** Factors associated with the occurrence of clinical pregnancy evaluated by binary logistic regression model.

Variable	OR (IC 95% OR)	*p*-value
**AGE RANGE** ≤ 35 years From 36 to 40 years ≥ 41 years	1.000 0.563 (0.438; 0.721) 0.319 (0.218; 0.462)	- **<0.001** **<0.001**
**EMBRYO TRANSFER STAGE** Cleavage Blastocyst	0.381 (0.298; 0.485) 1.000	**<0.001>** -
**FREEZING TIME** 0 to 90 days 91 to 180 days 181 to 360 days > 360 days	1.000 0.668 (0.500; 0.888) 0.836 (0.581; 1.195) 0.759 (0.550; 1.041)	**> - 0.006** 0.329 0.089
**Number embryos transfer**	2.737 (2.273; 3.313)	**<0.001**

*p*-value HL = 0.134.

### Factors associated with implantation rate

Table 3 presents the factors associated with implantation rates higher than 50% among patients who achieved clinical pregnancy (n=612). This threshold was used as the outcome it represents is a clinically meaningful implantation performance per embryo transferred. In the univariate analysis, statistically significant associations were found with maternal age (*p*<0.001), embryo stage at transfer (*p*<0.001), embryo freezing time (*p*=0.019), and number of embryos transferred (*p*<0.001).

**Table 3:** Factors associated with the occurrence of clinical pregnancy evaluated by binary logistic regression model.

Variable	Implantation rate		p-value
	Up to50% (n=451)	> 50% (n=161)	
	n (%)	n (%)	
**AGE RANGE** ≤ 35 years From 36 to 40 years ≥ 41 years	231 (51.2) 148 (32.8) 72 (16.0)	93 (57.8) 65 (40.4) 3 (1.9)	**<0.001^Q^**
**EMBRYO TRANSFER STAGE** Cleavage Blastocyst	310 (68.7) 141 (31.3)	40 (24.8) 121 (75.2)	**<0.001^Q^**
**FREEZING TIME** 0 to 90 days^*^ 91 to 180 days 181 to 360 days^*^ > 360 days^*^	252 (55.9) 77 (17.1) 52 (11.5) 70 (15.5)	110 (68.3) 26 (16.1) 12 (7.5) 13 (8.1)	**0.019^Q^**
**NUMBER EMBRYOS TRANSFER** 1 2 3 4	0 (0.0) 312 (93.1) 115 (84.6) 25 (96.2)	116 (100.0) 23 (6.9) 21 (15.4) 1 (3.8)	**<0.001^W^**

Q Chi-square test, W Wilcoxon Mann-Whitney test.

* Statistically significant difference in implantation rate compared to the 0-90 days group (Tukey post hoc test, *p*<0.05).

Higher implantation rates were observed among patients aged ≤35 years, who received embryos in the blastocyst stage, had freezing length ≤90 days, and had only one embryo transferred. Post hoc analysis using Tukey’s test identified statistically significant differences in implantation rates between the groups 0-90 days and 181-360 days (*p*=0.0187), as well as 0-90 days and >360 days (*p*=0.0001), suggesting a decline in implantation rate with longer embryo storage duration.

Table 4 presents the results of the multiple binary logistic regression performed to identify independent predictors of implantation rate >50%. The model included maternal age, freezing time (using corrected intervals), embryo stage at transfer, and number of embryos transferred. After adjustment, freezing time was not an independent predictor, with odds ratios of 1.258 (95% CI: 0.615-2.572; *p*=0.530) for 91-180 days; 0.949 (95% CI: 0.357-2.521; *p*=0.917) for 181-360 days; and 0.877 (95% CI: 0.328-2.346; *p*=0.794) for >360 days, compared to the reference group (0-90 days).

**Table 4:** Results of multiple binary logistic regression for predictors of implantation rate greater than 50% among patients who achieved clinical pregnancy (n=612).

Variable	OR (IC 95% OR)	p-value	95% CI Lower	95% CI Upper
**AGE RANGE** ≤ 35 years From 36 to 40 years > 41 years	0.287 1,000 0.310	<0,001 - 0.078	0.163 - 0.084	0.505 - 1.139
**EMBRYO TRANSFER STAGE** Cleavage Blastocyst	1,000	-	-	-
**FREEZING TIME** 0 to 90 days 91 to 180 days 181 to 360 days > 360 days	1,000 1.259 0.951 0.879	- 0.496 0.911 0.768	- 0.648 0.393 0.375	- 2.445 2.301 2.063
**NUMBER OF EMBRYOS TRANSFERED**	0.065	<0.001	0.039	0.11

In contrast, cleavage-stage transfers remained significantly associated with lower odds of implantation >50% (OR=0.406; 95% CI: 0.188-0.877; *p*=0.022). The number of embryos transferred was also inversely associated with the outcome (OR=0.066; 95% CI: 0.040-0.111; *p*<0.001). The ≤35 years age group was associated with lower odds of implantation >50% compared to the 36-40 years group (OR=0.287; 95% CI: 0.163-0.505; *p*<0.001), possibly reflecting more conservative clinical practices in patients with better prognosis. The ≥41 years group showed a trend toward lower implantation rates, although not statistically significant (OR=0.310; 95% CI: 0.082-1.176; *p*=0.085).

Due to the strong imbalance in implantation rates across embryo transfer groups, categorical modeling of this variable led to collinearity and convergence issues in the regression model. Therefore, the number of embryos transferred was retained as a continuous variable, ensuring statistical stability and better interpretability of the results.

## DISCUSSION

Our multicenter study involving data from 1,568 patients demonstrated that embryo storage affects clinical embryo implantation and clinical pregnancy rates. Embryos transferred within 90 days of vitrification showed higher implantation and clinical pregnancy rates when compared to embryos after 91-180 days, 181-360 days, and over 360 days. Patient age was also a relevant factor affecting success rates as women aged 35 or younger at the time of freezing had higher pregnancy rates compared to those aged 36-40 and 41 years or older.

Results shown here are in agreement with the study by Li *et al.* (2020), which used retrospective data from 24,698 patients undergoing freeze-all embryo transfer cycles to analyze the effects of storage duration after vitrification on pregnancy rates and neonatal outcomes in women. Their study demonstrated that pregnancy and implantation rates decreased with prolonged storage duration with no significant differences in neonatal outcomes, such as gestational age, birth weight, preterm birth, low birth weight, high birth weight, macrosomia and congenital defects.

Our research also aligns with the meta-analysis by Matorras *et al.* (2021), which analyzed 12 publications meeting inclusion criteria; totaling 17,948 frozen embryo transfers. Authors concluded that clinical pregnancy and biochemical pregnancy rates were slightly higher in immediate transfer groups compared to extended storage periods. However, no significant differences were observed in implantation rates.

In contrast, the study by Wirleitner *et al.* (2013) found no difference in clinical pregnancy rates after vitrified blastocyst transfer in relation to storage duration after prolonged storage. However, they only evaluated blastocyst-stage embryos and a smaller transfer cohort of 603 patients when compared to ours.

The study by Liu *et al.* (2014) analyzed 867 thawing cycles and found that storage duration did not have a significant effect on survival, blastomere damage rate, implantation rate, pregnancy rate, single birth weight, and live birth rate for embryos frozen at the cleavage stage. The study by Ueno *et al.* (2018) analyzed 8,736 cycles of autologous blastocyst transfer and also demonstrated that vitrification storage duration did not negatively affect pregnancy rates nor neonatal outcomes. However, this study was limited to patients aged 35 to 39, from a single center, with a diagnosis of tubal infertility and previous transfer failures.

The decline in pregnancy and implantation rates with increased storage duration may be explained by several factors: (1) disruption of cellular bioenergetic processes leading to mitochondrial damage; (2) interference with genomic integrity due to dynamic freezing and thawing processes involving various physical and chemical factors, such as changes in osmotic and hydrostatic pressure, pH alterations, intracellular content changes, and constant temperature fluctuations (Albertini & Olsen, 2013); and (3) increased DNA fragmentation due to free radicals and the toxic effects of cryoprotectants (Hosseini *et al.*, 2009).

Our results highlight that patient age at the time of freezing was an important factor for pregnancy and implantation outcomes. Patients aged 35 or younger at the time of freezing had higher pregnancy rates compared to those aged 36-40 and 41 years or older. A similar result was observed for implantation rates. The effect of age on implantation and pregnancy rates was further demonstrated by other studies. The study by Cimadomo *et al.* (2018) investigated the influence of maternal age at the time of oocyte and embryo freezing. The results highlighted that advanced maternal age is associated with a decrease in oocyte quality and implantation rate, resulting in lower pregnancy rates.

Teruya *et al.* (2006) analyzed data from 789 patients aged 23 to 49 years who underwent 2,355 cycles of controlled ovarian hyperstimulation for embryo transfer after IVF over a 15-year study period and concluded that pregnancy rates in embryo transfer cycles performed in patients between 23 and 46 years of age declined significantly with advancing patient age, with a steeper decline observed after age 35. The meta-analysis by Cil *et al.* (2013) analyzed 10 studies involving 2,265 oocyte freezing cycles from 1,805 patients and concluded that live birth success rates decreased with age, regardless of the freezing technique used.

Another relevant point demonstrated by our study was that embryo transfer stage is an important factor, indicating that thawed embryos transferred at the blastocyst stage have higher pregnancy and implantation rates compared to embryos transferred at the cleavage stage.

Our data are supported by the prospective study by Fernández-Shaw *et al.* (2015), which showed that blastocyst transfer appears to improve clinical pregnancy rates compared to cleavage-stage transfers, with more significant improvement observed in patients aged 35 years or older. The meta-analysis by Zeng *et al.* (2018), analyzing eight studies with more than 6,590 cycles, concluded that blastocyst transfer had a higher implantation rate than cleavage-stage embryo transfers, but there was no significant difference in clinical pregnancy rates.

The systematic review and meta-analysis by Martins *et al.* (2017), which analyzed 12 studies, found no superiority of blastocyst transfer compared to cleavage-stage embryo transfer in clinical practice concerning clinical pregnancy, cumulative pregnancy, and miscarriage rates. The influence of embryo stage at transfer remains a matter of debate. Further prospective studies are needed to understand the possible causes of reduced pregnancy and implantation rates after prolonged storage periods as well as the effects of freezing and thawing on embryos development and implantation potential.

The main limitation of our study was the retrospective approach used in data collection and analysis. Another limitation is that we evaluated the ongoing pregnancy rate rather than the live birth rate which would be a better indicator of success rates. While live birth rate is widely used as a success indicator in assisted reproductive technology, the ongoing pregnancy rate is often adopted as an alternative measure (Barnhart, 2014).

Strengths of our study include the significant number of patients as well as homogeneous clinical and laboratory practices that the clinics involved in the study adopted, such as the controlled ovarian stimulation protocols and laboratory routines. Additionally, rigorous inclusion and exclusion criteria were used which allowed us to minimize biases by excluding patients who did not meet the study criteria.

## CONCLUSION

Storage duration, patient age, and embryo stage may adversely affect FET outcomes. Women older than 35 years of age and embryos at the cleavage stage are at increased risk of lower pregnancy and implantation rates after extended storage.

Further studies involving other centers with larger numbers of participants are needed to establish the influence of storage time, embryo staging and female age on success rates in terms of pregnancy and implantation rates
